# Meganucleases and Other Tools for Targeted Genome Engineering: Perspectives and Challenges for Gene Therapy

**DOI:** 10.2174/156652311794520111

**Published:** 2011-02

**Authors:** George Silva, Laurent Poirot, Roman Galetto, Julianne Smith, Guillermo Montoya, Philippe Duchateau, Frédéric Pâques

**Affiliations:** 1 Cellectis Genome Surgery, 102 Avenue Gaston Roussel, 93 235 Romainville, Cedex, France; 2 Cellectis, 102 Avenue Gaston Roussel, 93 235 Romainville, Cedex, France; 3 Macromolecular Crystallography Group, Structural Biology and Biocomputing Programme, Spanish National Cancer Centre (CNIO), Melchor Fdez. Almagro 3, 28029 Madrid, Spain

**Keywords:** Homing endonuclease, Zinc-finger nuclease, Recombinase, Transposons, gene transfer, protein engineering, viral vector.

## Abstract

The importance of safer approaches for gene therapy has been underscored by a series of severe adverse events (SAEs) observed in patients involved in clinical trials for Severe Combined Immune Deficiency Disease (SCID) and Chromic Granulomatous Disease (CGD). While a new generation of viral vectors is in the process of replacing the classical gamma-retrovirus–based approach, a number of strategies have emerged based on non-viral vectorization and/or targeted insertion aimed at achieving safer gene transfer. Currently, these methods display lower efficacies than viral transduction although many of them can yield more than 1% engineered cells in vitro. Nuclease-based approaches, wherein an endonuclease is used to trigger site-specific genome editing, can significantly increase the percentage of targeted cells. These methods therefore provide a real alternative to classical gene transfer as well as gene editing. However, the first endonuclease to be in clinic today is not used for gene transfer, but to inactivate a gene (CCR5) required for HIV infection. Here, we review these alternative approaches, with a special emphasis on meganucleases, a family of naturally occurring rare-cutting endonucleases, and speculate on their current and future potential.

## INTRODUCTION TO TARGETED APPROACHES

### Different Strategies for Safer Gene Therapy

At the turn of the last millennium, the successful treatment of several X-SCID patients was a major milestone in the field of gene therapy [1, 2]. However, the importance of safer approaches has been emphasized by a series of severe adverse events (SAEs); e.g. the appearance of leukemia in X-SCID treated patients [3-5] and of myelodysplasia in a clinical trial for Chronic Granulomatous Disease (CGD) [6]. These SAEs were all associated with the integration of the transgene next to a proto-oncogene, and continued efforts have been devoted to the development of new, safer vectors. One such approach involves self-inactivating (SIN) gamma-retrovirus and lentivirus-based vectors, which when integrated lack enhancer and promoter sequences from the LTR [6-13]. These new vectors should decrease or alleviate the risks of proto-oncogene activation by insertional mutagenesis [14]. Meanwhile, these same SAEs fostered a growing interest for alternative strategies, which we will refer to as “targeted approaches” that consist of replacing random integration of therapeutic transgenes by targeted insertion or even correction of the deleterious mutation. Results are beginning to accumulate for clinical trials using new viral vectors [8, 15], and the first clinical trial using a targeted approach has been initiated [16]. Although additional time will be necessary to evaluate more precisely the potentials and limits of each method, targeted approaches remain today, at least conceptually, more attractive than classical gene transfer.

### Targeted Approaches: A Prosperous Methodology

Homologous gene targeting, first described in yeast by the laboratories of Gerald Fink [17], Jack Szostak [18-20] and, in its ultimate design, by Rodney Rothstein [21], was one of the first methods for rational genome engineering. This technique, successfully transferred to mammalian cells by the laboratories of Mario Capecchi [22-24] and Oliver Smithies [25-27], remains to this day a standard for the generation of engineered cells or knock-out mice [28]. An inherently low efficiency has nevertheless prevented it from being used as a routine protocol in most cell types and organisms. As homologous gene targeting can typically be observed in only 10^-6^ to 10^-9^ of treated mammalian cells [29], such frequencies did not obviate its use for gene and cell therapy. To address these issues, an extensive assortment of rational approaches has been proposed with the intent of achieving greater than 1% targeted modifications. The proven benefit of these emerging approaches lies in their ability to engineer the genome of a variety of cell types (including human stem cells). Nevertheless, researchers have invariably tried to assess their potential for therapeutic applications. In the therapeutic field, the replacement of classical gene transfer (resulting in random integration into the genome) with targeted gene insertion, or even targeted correction, has become a commonly accepted potential solution and thus the goal of many laboratories.

### Targeted Approaches Based on Recombinases

Several targeting strategies rely on site-specific recombination processes mediated by recombinases or transposases. Such methods have an inherent advantage since these proteins catalyze all the integration steps in a practically autonomous manner (e.g. transposases may require host factors). Their activity thus does not depend on the homologous recombination machinery. Homologous recombination is a complex maintenance system, and many studies argue that it is essentially active in the late S and G2 phases of the cell cycle [30-37]. As a consequence, the efficacy of recombinase or transposase-mediated integration should be relatively cell-type independent, whereas methods based on homologous gene targeting would be very inefficient in post-mitotic or quiescent cells.

The use of the Streptomyces phage ФC31 integrase to incorporate transgenes into the genome of mammalian cells provides an early example of a site-specific recombination approach. The ФC31 integrase is a serine recombinase that catalyzes the insertion of the phage genome into the bacterial genome via recombination between the phage attachment site (attP) and the bacterial attachment site (attB) [38, 39]. However, as pseudo integration sites can be found in mammalian cells, it was also possible to induce significant integrase-mediated insertion in specific loci in mammalian chromosomes [40]. In subsequent experiments, this technology was used to insert transgenes not only in a series of immortalized cell lines but also primary cells [41-44], including human embryonic stem (hES) cells [41, 45]. The system proved robust enough for *in vivo* use, wherein co-injection of a ФC31 integrase expression cassette with a plasmid carrying an attB site resulted in high frequencies of integration coupled with stable transgene expression in various tissues, such as mouse muscles [46, 47], rabbit joints [48], rat retina [49] and mouse liver [50, 51]. Using hydrodynamic injection, Olivares and colleagues observed integration in liver tissue at two major sites, one of which had detectable incorporation of a Factor IX transgene in 0.6 to 3.6% of total hepatocytes [51]. This strategy is limited, however, by an inability to choose the integration site. Whereas the most favored unique landing sequence occurred at chromosome 19q13.31, circa one hundred other integration sites have been identified [52]. Recently, mutated derivates of ФC31 with enhanced efficiency and specificity have been reported [53], offering great promise for success in the near term.

The Cre and FLP tyrosine recombinases have also been envisioned as reagents to mediate targeted insertions. These proteins have been widely used in genome engineering experiments, albeit always to recombine transgenes carrying their respective target sequences. To this end, several attempts have been made to redesign recombinase site-specificity [54-57], the most remarkable involving a modified recombinase able to recognize sequences from the HIV1 virus and used to excise a provirus from the human genome [54]. Whereas these studies were based on the redesign of the recombinase substrate specificity, an alternative strategy was used by other groups who fused the catalytic domain of the Tn3 resolvase or the Gin recombinase to a Zinc-finger-based DNA-binding domain [58-60]. However, in these initial proof-of-concept designs the recombinase catalytic domain was still contributing to target preference. Using additional engineering steps, Gersbach and colleagues could suppress the target preference of the Gin moiety, thus producing a chimeric recombinase having essentially the selectivity of the chosen Zinc-finger DNA-binding domain [61].

### Targeted Approaches Based on Transposons

The stable introduction of therapeutic transgenes into human cells can be accomplished by transposon-mediated gene transfer and has been proposed as an alternative to viral-mediated gene delivery [62-64]. As transposases can efficiently catalyze gene transfer, transposition has long been used in Drosophila [65] to create transgenics. They have also widely been used in other organisms such as plants, fish, bacteria and others [66-68] in mutagenesis experiments. The inability of transposons to introduce a cargo of genetic material into a cell in an autonomous manner is both an advantage and a drawback: unlike viruses they do not present risks of lateral transfer yet one needs to use them in association with a vector to introduce them into the cell. Several transposons have nonetheless been used in gene transfer experiments in vertebrate cells. The most advanced system is perhaps that derived from the Sleeping Beauty (SB) transposon, an ancient mobile genetic element in fish that could be modified to sustain elevated levels of activity in mammalian cells, with up to 5-10% integration [69, 70]. In similar experiments, transposons based on the Piggy Back system were used to make induced pluripotent stem (iPS) cells [71, 72]. Importantly, the efficacy of transposase-mediated gene insertion can be extremely high, and as a consequence high rates of gene transfer can be observed even with a suboptimal vectorization method. For example, the association of electroporation methods and of the SB100X enhanced transposase results in an efficacy of gene insertion of up to 50% in CD34+ blood cells [70]. Transposons remain non-targeted gene transfer vehicles, and as such several attempts have been made to endow them with sequence specificity by fusion with a DNA binding domain. Initial attempts with the SB transposon resulted in an enrichment for targeted integration in human cells [73]. More recently, the ISY100 transposase from a cyanobacteria transposon was fused with a Zinc-finger DNA binding domain (Zif268) and used to efficiently target a specific site in *E. coli.*[74]. While promising as a proof-of-concept, the activity of this transposon in eukaryotic cells is unknown. In terms of production, one of the major drawbacks of viral vectors is the need for a tedious manufacturing process, a difficulty compounded in the case of therapeutic applications where Good Manufacturing Practice (GMP) is compulsory. The ability to achieve similar efficacies while circumventing these issues is a significant plus for transposon-based approaches. A clinical trial using SB-mediated transfer of a chimeric antibody receptor (CAR) for the treatment of leukemia has recently been approved [75, 76].

### Targeted Approaches based on Homologous Recombination

In parallel to the development of methods based on site-specific recombination, many groups have focused on enhancing the efficacy of homologous gene targeting. Two major disciplines have become apparent: (i) methods that we will call “matrix optimization”, essentially consisting of modifying the targeting vector structure to achieve maximal efficacy, and; (ii) methods involving additional effectors to stimulate homologous recombination (HR), generally sequence-specific endonucleases. The field of matrix optimization has covered a wide range of techniques, with varying degrees of success. For example, attempts to replace the DNA repair matrices used in classical gene targeting experiments by chimeric DNA-RNA modified oligonucleotides, including various modifications and/or specific secondary structures, were initially seen as promising yet eventually proved inefficient in many experiments [77].

Another type of optimization, Small Fragment Homologous Replacement (SFHR), relies on information exchange between short DNA fragments homologous to the targeted sequence and the endogenous locus [78]. The small size of the DNA fragment limits this approach to gene correction and does not allow for gene insertion or gene replacement experiments. All the same, SFHR was successfully applied to correct various mutations associated with monogenetic disorders. Very different frequencies have been reported, and a number of publications report correction of the human HPRT, CFTR, HBB and DMD genes in cell lines and primary cells, with frequencies in the range of 1% or even 10% of transfected cells [78-80]. A recent report describes correction of the CFTR gene in up to 60% of mouse ES cells [81].

The use of Adeno-Associated Virus (AAV) as vectors for the repair matrix provides another way to increase HGT rates. Homologous gene targeting between an AAV vector and a chromosomal sequence can occur at frequencies in the range of 0.1-1.4 % in a variety of cell lines [82-84]. AAV-mediated gene inactivation was also used to disrupt the COL1A1 and COL1A2 genes in human mesenchymal stem cells (MSCs) [85, 86] with an efficacy of 0.06-0.23% per treated cell for COL1A1. In addition, AAV-mediated gene targeting has been used to correct a LacZ marker at a frequency of 0.1% *in vivo* in a mouse model [87]. Research continues towards optimized matrices, although as noted in the next section the use of effectors to stimulate HR is becoming more widespread.

Since the first reports of using the I-SceI meganuclease in mammalian cells, the practice of stimulating HR via nucleases has become standard in many fields [88, 89]. I-SceI itself has been applied to numerous experimental designs in a variety of cell types and organisms (see below, “Use of meganucleases for genome engineering”). In addition to natural endonucleases, other sequence specific nucleases have been investigated. Endonucleases for genome engineering have been classified into three groups by Pingoud and Silva [90]: chemical nucleases, ZFNs and meganucleases. This classification should be updated given the recent emergence of a fourth category, the TALE nucleases (see below).

Chemical endonuclease are synthetic endonucleases resulting from the fusion of DNA-reactive agents to a DNA-binding polymer, such as triplex-forming oligonucleotides (TFO) and can in theory provide a straightforward solution (for review see [91]). Because the sequence specificity of such polymers can be designed *a priori*, it eliminates the extensive screening process usually necessary for the identification of a new peptidic DNA-binder. A variety of active domains such as a topoisomerase inhibitor (camptothecin) [92], psoralen [93], bypiridine [94], or a restriction enzyme [95] in fusion with TFO have been described as potential sequence-specific tools for genome engineering. While in principle promising, intracellular delivery, efficiency of endogenous gene modifications as well as safety issues still need to be addressed for consideration in therapeutic applications.

To alleviate the delivery and safety issues associated with chemically-derived nucleases, attempts have been made to engineer protein endonucleases with programmable specificities. Among the most prevalent contenders are the Zinc-finger nucleases (ZFN), generated by fusing Zinc-finger-based DNA binding domains to an independent catalytic domain via a flexible linker [96-98]. The archetypal ZFNs are based on the catalytic domain of the Type IIS restriction enzyme FokI and have been successfully used to induce gene correction, gene insertion, and gene deletion. These studies have been reviewed by others [88, 99-102] and will not be described in detail. In summary, several groups have reported targeted HR events occurring at frequencies greater than 1% (and up to 50%) in immortalized human cell lines as well as primary cells including ES cells [103-109]. However, low efficacies have been reported in human ES, iPS [108] and hematopoietic stem cells (HSCs) [103]. Zinc Finger-based DNA binding domains are made of strings of 3 or 4 individual Zinc Fingers, each recognizing a DNA triplet [110]. In theory, one of the major advantages of ZFNs is that they are easy to design, using combinatorial assembly of preexisting Zinc Fingers with known recognition patterns [111-113]. However, close examination of high resolution structures shows that there are actually cross-talks between units [114], and several methods have been used to assemble ZF proteins by choosing individual Zinc Fingers in a context dependant manner [106, 115-117] to achieve better success rates and reagents of better quality.

Recently, a new class of chimeric nucleases using a FokI catalytic domain have been described [118, 119]. The DNA binding domain of these nucleases is derived from Transcription Activator Like Effectors (TALE), a family of proteins used in the infection process by plant pathogens of the *Xanthomonas* genus. In these DNA binding domains, sequence specificity is driven by a series of 33-35 amino acids repeats, differing essentially by two positions [120, 121]. In the DNA target, each base pair is contacted by a single repeat, the specificity resulting from the two variant amino acid of the repeat. The apparent modularity of these DNA binding domains has been confirmed to a certain extent by modular assembly of designed TALE-derived protein with new specificities [120, 121]. However, one cannot yet rule out a certain level of context dependence of individual repeat/base recognition patterns, as was observed for Zinc Finger proteins (see above). TALE-nucleases have been shown to be active in a cell-based assay in yeast [118, 119], and their exact level of engineerability, activity and specificity remains to be explored. Nevertheless, the emergence of this promising new family of endonucleases highlights the fact that the list of reagents continues to grow. The FokI effector appears capable of being fused to any convenient DNA binding domain, and the creation of functional I-SceI::FokI fusions [122] illustrates this potential diversity.

While ZFNs have shown considerable promise, there has been a continued interest in the natural meganucleases given the extensive use of I-SceI by a large community of scientists. Indeed, I-SceI has been used since the early nineties for genome engineering experiments and remains today the reference (“gold standard”) in the field with regard to activity and specificity. As the meganuclease family of which I-SceI is a member includes hundreds of other natural proteins, a special section has been included below (see “The Meganuclease Family”).

### Targeted Approaches based on Non-Homologous End-Joining

In addition to the HR pathway for repairing double-strand breaks (DSBs) in DNA, an error-prone DNA repair mechanism called non-homologous end joining (NHEJ) has been identified [123]. NHEJ seems to comprise at least two different components: (i) a pathway that consists mostly in the direct re-joining of DSB ends, and which depends on the XRCC4, Lig4 and Ku proteins, and; (ii) an alternative NHEJ pathway, which does not depend on XRCC4, Lig4 and Ku, and is especially error-prone, resulting mostly in deletions, with the junctions occurring between micro-homologies [124-129].

For DSBs induced by biological reagents, *e.g.* meganucleases and ZFNs, which cleave DNA by hydrolysis of two phosphodiester bonds, the DNA can be rejoined in a seamless manner by simple re-ligation of the cohesive ends. Alternatively, deleterious insertions or deletions (indels) of various sizes can occur at the breaks, eventually resulting in gene inactivation [16, 106, 111, 130-135]. The nature of this process, which does not rely on site-specific or homologous recombination, gives rise to a third targeted approach based on endonuclease-induced mutagenesis. This approach, as well as the related applications, may be simpler than those based on homologous recombination in that (a) one does not need to introduce a repair matrix, and; (b) efficacy will be less cell-type dependant (in contrast to HR, NHEJ is probably active throughout the cell cycle [35]).

Targeted mutagenesis based on NEHJ has been used to trigger inactivation of single or even multiple genes in immortalized cell lines [136, 137] . In addition, this method opens new perspectives for organisms in which the classical HR-based gene knock-out methods have proven inefficient, or at least difficult to establish, such as rat [138-140], fish [131] and plants [106, 134, 141, 142]. Interestingly, the first clinical trial launched with ZFNs relies on ZFN-mediated mutagenesis of the human CCR5 gene [16] in order to block HIV1 entry into T-cells in AIDS patients (see below, “Targeted mutagenesis of human genes”).

## THE MEGANUCLEASE FAMILY OF ENDONUCLEASES: PROPERTIES AND USE

As outlined previously, endonucleases for genome engineering can be classified into four groups: chemical nucleases, ZFNs, meganucleases [90], and now, TALE-nucleases [118, 119]. This section focuses on meganucleases, which have been used for more than 15 years to induce gene targeting. Recent advances in re-engineering meganuclease specificity have further enhanced their scope of application. The scores of publications and contributions to scientific meetings related to meganucleases reveal a significant evolution in adoption and applicability, with a growing community finding an interest in the exceptional properties of these proteins.

### The Meganuclease Family

Meganucleases, also called homing endonucleases, can be divided into five families based on sequence and structure motifs: LAGLIDADG, GIY-YIG, HNH, His-Cys box and PD-(D/E)XK [143, 144]. The most well studied family is that of the LAGLIDADG proteins, which have been found in all kingdoms of life, generally encoded within introns or inteins although freestanding members also exist. To date, a purposeful role within the host has not been identified for these proteins and they tend to be classified as “selfish genetic elements”. With few exceptions, LAGLIDADG proteins exhibit one of two primary activities: (a) they function as RNA maturases involved in facilitating the splicing of their own intron, or; (b) they function as highly specific endonucleases capable of recognizing and cleaving the exon-exon junction sequence wherein their intron resides, thus giving rise to the moniker “homing endonuclease”. It has been hypothesized that homing endonucleases have a so-called “life-cycle” [145] as illustrated in Fig. (**1**) (i) first, they start out as invasive endonucleases capable of mobilizing their coding sequence; (ii) upon “invasion”, they acquire a concomitant RNA maturase activity to help ensure proper splicing of their intron; (iii) over time, the “invasive” nuclease activity is lost, leaving only the RNA maturase function, and (iv) finally, upon losing the maturase activity, propagation of the intron becomes unviable and the intron is lost. It is thus inferred that the functionality of a given LAGLIDADG protein (endonuclease, maturase, or both) represents a snapshot into the current state of its lifecycle.

### Structure of LAGLIDADG Endonucleases

A considerable body of both biochemical and genetic work had established that LAGLIDADG homing endonucleases could be used as molecular tools. It had long been known that the defining sequence motif, LAGLIDADG, represented an essential element for enzymatic activity. Some proteins contained only one such motif, while others contained two; in both cases the motifs were followed by ~75-200 amino acid residues having little to no sequence similarity with other family members. In 1997, two groups shed light on the organization of LAGLIDADG proteins with the structure of both a single-motif protein (I-CreI) [146] and an intein-encoded double-motif protein (PI-SceI) [147]. In both cases the endonuclease domain adopted a similar αββαββα fold, with the LAGLIDADG motif comprising the terminal region of the first helix and not only contributing to a bipartite catalytic center but also forming the core subunit/subunit interaction. Two such α/β domains assemble to form the functional protein, with the β-strands in each creating a saddle-shaped DNA binding region. That two such subunits were needed for function could be definitively established, as exemplified by I-CreI, a homodimer, having an overall architecture similar to that of the double-motif endonuclease domain of PI-SceI. The α/β fold is a hallmark of LAGLIDADG proteins and has since allowed for better homology modeling and structure/function prediction for proteins lacking structural data.

It has been estimated that LAGLIDADG proteins specifically recognize a DNA sequence ranging from 14 to 40 base pairs in length. Although the initial structures were solved in the absence of target DNA, several labs were able to successfully exploit the structural data to better understand protein-DNA interactions. First, it was clear from the PI-SceI structure that the additional splicing domain characteristic of intein-encoded proteins was flanked by a region capable of specifically contacting DNA [148, 149], a concept borne out when the DNA co-crystal structure was finally solved [150]. These studies addressed the co-evolution of the endonuclease domain (domain II) with the surrounding splicing domain (domain I). Second, having structural data allowed for better predictions regarding studies aimed at investigating the determinants of specificity. Whereas it had previously been known that most homing endonucleases could tolerate single-base changes throughout their recognition sequence, site-sequence degeneracy studies could be rationalized (I-CreI [151], PI-SceI: [152]). Nevertheless, it was only a short time later that DNA co-crystal structures were available, giving not only the first glimpse of LAGLIDADG protein-DNA contacts (I-CreI [153]) but also enabling theories about the nature of catalysis [154, 155]. Despite each subsequent co-crystal structure adding individual idiosyncrasies to the overall picture of LAGLIDADG protein-DNA interactions [150, 155-162], several generalities persist: (i) specificity contacts arise from the burial of the extended β-strands into the major groove of the DNA, with the DNA binding saddle having a pitch and contour mimicking the helical twist of the DNA; (ii) although extensive, the complete complement of hydrogen bonding potential between the protein and DNA is never fully realized, with many contacts being water-mediated; (iii) cleavage to generate the characteristic 4-nt 3’-OH overhangs occurs across the minor groove, wherein the scissile phosphate bonds are brought closer to the protein catalytic core by a distortion of the DNA in the central “4-base” region; (iv) cleavage occurs via a proposed two-metal mechanism, sometimes involving a unique “metal sharing” paradigm; (v) and finally, additional affinity and/or specificity contacts can arise from “adapted” scaffolds, in regions outside the core α/β fold.

On first inspection it appears that these generalities could define a fixed rule-set, which could help manipulating LAGLIDADG protein-DNA interactions. However, several studies have shown that related proteins can use different subsets of residues to recognize similar DNA [158, 163]. As discussed below, this “relaxed” property of meganucleases has proved to be a double-edge sword, making it at once both easy and problematical to re-engineer specificity.

### Engineering Meganucleases

The meganuclease field has recently exploded with novel and interesting engineering applications. In a “bulk approach”, several teams tested the concept of subunit interchangeability by fusing both similar and disparate α/β domains, giving rise to hybrid proteins having hybrid specificity derived from each half of the parental protein targets [157, 164, 165]. These proof-of-concept studies addressed not only the feasibility of a “mix and match” approach but also the evolutionary concept that double-motif LAGLIDADG proteins arose from a gene duplication event. Gene fusion alleviates the restriction imposed by homodimers to symmetric or pseudo-symmetric targets by allowing for asymmetric mutations in each subunit and thus recognition of asymmetric DNA targets. This model has undeniably proved valuable in paving the way for selection and screening studies based on the independence of DNA recognition by individual α/β subunits.

Several strategies for engineering meganucleases involve a semi-rational approach in which specific residues are mutated on the basis of prior structural or functional knowledge to create libraries with limited diversity. Using I-CreI as a model scaffold, several groups have been able to alter its DNA recognition properties both on a small and large scale in terms of total proteins re-engineered. Initial successes with the I-CreI [166-168] and I-SceI scaffolds [169, 170] resulted in the identification of only a few mutants due to the limited screening technologies. To analyze a much larger number of mutant/target combinations, an automated high-throughput screening method was used for the I-CreI scaffold, resulting in the identification of hundreds of mutants with locally altered specificities [171, 172]. These variants were shown to maintain the essential properties of the initial scaffold, i.e. proper folding and stability, cleavage efficiency and a narrow specificity. This has since led to a combinatorial approach in which different sets of I-CreI mutants are combined in the same protein, resulting in entirely redesigned meganucleases cleaving chosen sequences [172, 173].

Currently, engineered meganucleases cleaving two different human genes, XPC and RAG1 [172, 173], as well as a meganuclease cleaving a maize genomic sequence [142], have been described in the literature. Additionally, continued engineering efforts have allowed the production of custom meganucleases cleaving several dozens of chosen natural sequences (our unpublished data). While on the surface these studies solidify the practicability of altering meganucleases, bona fide engineering of specificity has proved to be more complex. Progress remains to be made if one wants to use the full potential of other LAGLIDADG proteins, including natural single-chain variants. Detailed studies of the LAGLIDADG scaffold have demonstrated noteworthy pitfalls, including differences in how each subunit interacts with DNA, either in a homodimer context to efficiently bind asymmetric DNA [162] or within the same monomer, displaying a disparity in affinity vs. specificity [174]. Moreover, regions outside the core fold have been implicated in being important for target site DNA interactions [175]. Finally, the homodimer nature of the proteins themselves creates an inherent impasse when designing proteins for asymmetric targets, as three species of functional enzyme will arise in the mix.

Fortunately, the future of meganuclease engineering is quite promising as all of these challenges are being overcome and in the process innovative studies have shed light on new and exciting avenues. Comprehensive computational studies have given rise to not only specificity re-engineering [176], but have also addressed the dimerization issue via targeting protein-protein interactions of the subunits [177] as well as through mimicking nature with single-chain designs [178, 179]. Exhaustive analysis of the I-AniI scaffold [180, 181] has led to better computational methods that can exploit binding energy for designs [180]. Through both computational [182] and structural [161] analysis of novel structures of engineered proteins in complex with DNA, insight has been gained into the intricacies of the protein-DNA contacts that appear to involve substantial conformational changes in both the protein and/or DNA; findings that suggest specificity engineering is more than a point-by-point process Fig. (**2**). Moreover, new opportunities are being explored through the generation of targeted “mega-nickases” (I-SceI [183], I-AniI [184]) that can in principle provide similar levels of induced HR with a minimization in the frequency of NHEJ.

### Use of Meganucleases for Genome Engineering

DNA damage is a naturally occurring event that can lead to chromosomal aberrations or cell death. DSBs are particularly hazardous to the cell as they can potentially lead to genome rearrangements. Not surprisingly, a variety of repair strategies have evolved to ensure genomic integrity. As outlined above, these processes can be either error-prone (NHEJ) or conservative (HR), depending on the mechanisms involved. Understanding the nature of each mechanism can be crucial in the context of gene targeting. Namely, from the genome engineering point of view, the challenge of using the cellular DNA repair machinery lies in creating a precise DSB in a genome.

I-SceI is the prototypical meganuclease used for genome engineering. In nature, the protein stimulates HR by creating a site-specific DSB in the genome, in a process called homing (see above). The discovery of this function in the yeast *Sacharomyces cerevisiae* launched a new era in gene targeting [185]. Researchers studying DNA repair in mammalian cells soon realized the potential of such a tool. In the nineties, pioneering work used a chromosomal neomycin resistant gene interrupted by an I-SceI recognition site as a reporter to monitor gene correction events in mouse cell lines upon introduction of an I-SceI expression vector and a DNA repair matrix. Gene correction could be achieved at frequencies of 3x10^-5^ in ES [186] and 4x10^-4^ in NIH3T3 [187] cells. Further experiments in NIH3T3 and PCVC7 cell lines were able to show that targeted gene insertion could also be obtained using this approach [188, 189]. In contrast to cell populations that received the DNA repair matrix alone, where no targeted events could be detected, frequencies of 1.8x10^-4^ to 4x10^-4^ targeted events per transfected cell could be achieved. Later studies were able to realize an even higher frequency of gene targeting. For example, Donoho and colleagues obtained nearly 1% recombination in ES cells [190], while Szczepek and coworkers could attain up to 10% with I-SceI (used as positive control) in 293 cells [191]. These analyses paved the way for the DSB-induced gene targeting technology, as they demonstrated a >1000-fold stimulation of events attributable to the activity of the meganuclease. Since then, this simple system has been widely adopted by the scientific community and has allowed for a better understanding of the DNA repair mechanism in a variety of cell types and experimental conditions [89].

Despite the notion that HR precisely repairs the template, in certain cases imperfect targeting events were reported, the consequences of which would be notable for therapeutic applications [187] [186]. These results highlighted the necessity to understand the intricacies of HR. For example, it was found that HR can occur at one end of the DNA junction while non-homologous recombination is carried out at the other. In a similar vein, the possibility to correct (or introduce) a point mutation at a distance from the DSB was studied. It was determined that the double-strand break repair (DSBR) pathway can result in the conversion of sequences adjacent to the DNA break, wherein the efficiency of the conversion decreases as the distance from the DSB increases. Elliott and colleagues observed a very sharp decrease of the conversion efficiency: a polymorphism located at a distance of circa 100 bp from the DSB was corrected in only 13-16% of the repair events, and by 400 bp correction dropped to only 3%. [192]. Meanwhile, Donoho and coworkers reported longer conversion tracts, with up to 13% correction at a distance of about 4000 bp from the DSB [190]. Finally, experiments in the human cell line 293H have shown that a mutations located 265 bp from the DSB could be efficiently (66%) corrected [193]. Despite the apparent discrepancies in these results, most likely arising in part from experimental design differences, the trend in conversion efficiency observed as a function of distance remains an important point to consider for any gene targeting approach.

Ultimately, the studies made with the I-SceI system to decipher the cellular mechanisms of DSBR have laid the foundation for all the main strategies envisioned today for genome engineering purposes (Fig. (**3**)). Gene correction [194, 195], gene insertion [189, 190, 196], and gene inactivation through a NHEJ mechanism [130] as well as the elimination of a DNA sequence by single-strand annealing (SSA) assays between direct repeats [130, 190, 197] were all addressed during these early studies. This last approach appears highly efficient as I-SceI is able to induce recombination between direct or inverted repeats at frequencies of up to 10^-1^ events among transfected mammalian cells. A similar frequency was obtained *in vivo* after tail-vein injection of an adenovirus expressing I-SceI into transgenic mice carrying a LacZ-based reporter gene: up to 1.3% of the hepatocytes showed recombination events [198]. Although cell-line development for protein production does not represent the focus of this review, it is worth mentioning that genes inserted by meganuclease-induced recombination show reproducible expression levels among targeted cellular clones [199, 200].

The I-SceI-based HR system has also been used to address fundamental questions that in many ways also gauge the limits of the methodology. For instance, the possibility of utilizing the homologous chromosome or an ectopic chromosomal locus as a repair template for I-SceI induced DSBR was investigated using cultured cells [201, 202]. In terms of gene therapy, this strategy would provide a tremendous advantage in the correction of mutations responsible for dominant monogenetic disease as the introduction into cells of the meganuclease could alone lead to gene correction. Unfortunately, when sequences are located on separate homologous or heterologous chromosomes, the recombination efficiency is extremely low (10^-6^-10^-5 ^events among transfected cells), making this approach impractical for therapeutic applications. Experiments using the template-based method have nevertheless shown that, in addition to mammalian cells, I-SceI-mediated recombination is robust and efficient in diverse organisms. It has been successfully used to induce various modifications such as mutagenesis, recombination between repeats or gene targeting in bacteria [203-207], mosquito [208, 209], fly [210] and plant [134, 211-216]. I-SceI has also been used to improve transgenesis efficiencies in various organisms such as frog [217], fly [218], fish [219], and sea anemone [220].

An obvious drawback to the use of natural meganucleases lies in the need to first introduce a known cleavage site into the region of interest. Although so-called pseudo-sites within a genome can be exploited by certain methods (e.g. the ФC31 integrase), such approaches are not always possible in therapeutic cases where targeting can demand a more stringent level of precision. To this end, engineered meganucleases are poised to address the targeting issue and should allow for a broader use of HR-based technology. Extensive characterization of an engineered I-CreI derivative cleaving the human RAG1 gene [178] has indeed demonstrated that a fully redesigned meganuclease can compare to I-SceI in terms of both efficacy and specificity. This RAG1 endonuclease could be used to induce 6% recombination in 293 cells and its relative specificity was comparable to that of I-SceI [178]. Similar results (1 to 20% targeted recombination) have been obtained in transfection experiments using other immortalized cell lines, not only with the RAG1 meganuclease but with many other I-CreI-derived engineered meganucleases tailored to uniquely cleave diverse targets in the human genome (our unpublished data).

## DIFFERENT STRATEGIES FOR GENE THERAPY BASED ON ENDONUCLEASES

In this section, the major strategies envisioned for the therapeutic use of rare-cutting endonucleases are outlined.

### Gene Correction Approaches

Several strategies exist for choosing the nature of the meganuclease-induced modification as well as the design of the targeting construct. Meganucleases can in principle be used to accomplish what may be considered as “ideal” gene therapy: true reversion of the deleterious mutation. This approach has practical limitations, since the efficacy of correction decreases rapidly as the distance from the initial DNA double-strand break increases. As a consequence, this approach can only be considered in diseases caused by a prevalent (and, in theory, targetable) mutation, such as sickle cell anemia [221]. An alternative approach along the same lines could involve inserting the whole coding sequence upstream of a deleterious mutation. In this case, although regions of the damaged gene would remain, they would be effectively “silenced” by the newly inserted corrected gene.

Gene repair strategies can more easily be envisioned wherein the particular disease itself presents a selective advantage upon treatment by gene therapy, such as SCID [222] or Wiskott-Aldrich syndrome (WAS) [223] . Endonucleases targeting two different genes involved in SCID diseases have been described to date: (i) the IL2RG gene that has been targeted by a ZFN [104, 105], and; (ii) the RAG1 gene, for which a meganuclease has been re-engineered to target [172, 178]. *In vivo*, there is a selective advantage for cells with a functional gene because signaling through the γc cytokine receptor or possessing a functional RAG1 recombinase confers a survival and/or proliferation signal during differentiation of lymphocytes [222, 224]. The IL2RG ZFN has been used in a variety of immortalized and primary cells. An initial design was used to achieve gene targeting in 18% of treated K562 cells, and 5% of treated T cells [104]. Using the latest ZFN variants engineered for minimal toxicity [105], 5-15% gene insertion has been observed in transfection experiments in K562 cells [109]. The use of non-integrative lentiviral vectors resulted in up to 6% of targeted insertion [103]. Likewise, with the meganuclease targeting the RAG1 gene, aiming at the same correction approach, up to 6% of targeted modifications could be observed in transfection experiments using 293 cells [178]. For comparison, the RAG1 meganuclease and the IL2RG ZFN appear to yield the same frequencies of homologous gene targeting in 293 cells (our unpublished data). In principle, these frequencies should be enough for an *ex vivo* treatement of SCID diseases, provided one can achieve them in hematopoietic stem cells (HSCs). However, achieving high efficiencies in HSCs might be problematic (see below, THE EFFICACY AND SAFETY ISSUES), and future studies will tell whether the quality of the nucleases reported above will be high enough to compound the intrinsic difficulties linked with the handling of human HSCs.

Other nucleases have been engineered to target genes whose correction by homologous gene targeting could be of therapeutic relevance. For example, a recent report describes a ZFN targeting the human PIG-A gene, involved in paroxysmal nocturnal hemoglobinuria (PNH) [108]. This ZFN allowed for gene targeting of 3% of transfected 293 cells, but these figures decreased by at least 100 fold in stem cells ([108]. Two meganucleases targeting the human XPC gene have also been described, but these proteins were tested only with reporter systems [161, 173], with targeting frequencies between 0.1% and 1% (our unpublished data). With these frequencies, the use of such nucleases will depend on selection procedures to sort targeted events. For therapeutic purposes, the selection scheme should be both (**a**) as non-invasive as possible, and; (**b**) at the expense of the multipotency or progenitor capacity of the treated cells.

### Insertion of Therapeutic Genes into a Safe Harbor

An increasingly popular approach in gene therapy today seems to be the targeted insertion into a so-called “safe harbor”. This strategy consists of introducing genetic material into a predefined locus unrelated to the disease-causing gene. In contrast with true gene correction, targeted insertion is not limited to the use of methods based on homologous gene targeting, and targeted recombinases or transposases could perform as well.

For this method to be valid, the locus of interest must possess two essential properties. First, an adequate safe-harbor locus needs to support sufficient and stable gene expression in the modified cells. For the gene therapy to be effective, the inserted element of interest must be available in the host at functional, if not “wild-type”, levels. Second, and most importantly, the safe-harbor locus needs to be “safe” in terms of not interfering with normal cell function and gene regulation. Adverse events could be caused by the deregulation of other genes, either by repression or induction of gene expression, or by inducing chromosomal instability. The difficulty in the safe-harbor approach stems from the ability to predict that a particular locus is safe, and many different strategies are currently being explored. For example, an ideal site for transgene expression was identified in mouse genetics studies of the ROSA26 locus, where insertion of a coding sequence leads to its constitutive expression without altering viability [225]. However, there is no evidence that the homologous human locus is similarly “safe”. A promising safe harbor is the AAVS1 locus, the most common integration site for AAV, a parvovirus lacking a known associated pathology [226]. AAVS1 is located on chromosome 19 within intron 1 of the PPP1R12C gene, and has been shown to allow inserted exogenous DNA to be transcribed [226, 227]. ZFNs targeting AAVS1 have recently been developed and used to induce targeted insertions in immortalized cell lines and primary cells, with stable expression of the transgene [107, 228]. In transfection experiments, frequencies of targeted events could reach circa 10% in K562 cells, 3% in HEK293 and Hep3B cells, and similar values in other cell lines. Gene targeting could also be observed in human stem cells, e.g. ES and iPS cells. However, targeted events were identified after selection for transformants having integrated a reporter gene placed in the repair matrix. Whereas targeted events represented a large fraction of the integrations (up to 61%), it is difficult to infer the absolute rate of gene targeting (e.g. the ratio of targeted events per transfected cell) in these studies [107, 228]. Nevertheless, on can suspect that it is low, which again raises efficacy issue for real-world therapeutic applications. If the use of a selection scheme has to be envisioned, it is with caution, for it would have to be non invasive and not affect the multipotency of the targeted stem cells. Future studies should tell whether the current targeting protocols can avoid selection procedures that might prove problematic.

While the above examples illustrate active research on potential safe-harbors, the choices of loci in these cases were fortuitous. However, given the constant progress in genome annotation, the mapping and description of multiple features (e.g. GC-clusters, non-coding RNA genes, sequence variations, chromatin-immunoprecipitation, mRNA expression pattern, etc) are available from publicly accessible databases, allowing for semi-rational selection of safe harbor loci. Perhaps the simplest way to keep cellular expression profiles unaltered is to target loci distal from functional genes, with an emphasis on avoiding tumor suppressor genes. While basic in concept, this strategy does not fully address the issue of effects on chromatin remodeling or chromosomal stability. The cell type that needs to be targeted must also be taken into account when investigating the usefulness of a particular locus. For example, a locus that supports stable gene expression in hematopoietic stem cells might not provide the same expression in neurons or hepatocytes. This discrepancy may arise from many factors, one of which being the variability in whether a safe locus is indeed “safe” in different cell types. In all cases, it must be noted that the choice of a viable safe harbor is coupled to its ability to be efficiently targeted, either by preexisting or engineered nucleases. Thus, beyond the issues of safety and stable gene expression, safe harbor accessibility must be considered in light of nuclease targeting potential, which could vary along the genome and between cell types, due to epigenetic modifications (there is at least one example in plants of a nuclease targeting two exactly identical sequences with different efficacies, these two different sequences being found in two different loci, in the SurA and SurB genes [106]). Despite these technical obstacles, the identification of a good safe-harbor/nuclease pair appears to be a readily attainable goal. Notably, an additional advantage of using a safe harbor strategy is that it can also be utilized in multiple indications as different genes can be expressed from the same locus.

### Targeted Mutagenesis of Human Genes

The use of meganucleases to target a mutagenic event at a specific locus is actively being investigated for numerous purposes. As evidenced by its widespread use in model systems, targeted mutagenesis can also have therapeutic perspectives. The selective disruption to restore the wild-type phenotype of mutated alleles in dominant diseases remains a challenge: an endonuclease able to discriminate the mutated allele from that of the wild-type is required. Whereas the treatment of monoallelic diseases by targeted mutagenesis represents an interesting concept, more practically this approach can be applied to the inactivation of unwanted genetic elements.

ZFNs have recently been used to inactivate the CCR5 gene, a co-receptor for HIV entry, leading to resistance against HIV infection [16]. Inactivation of 50% of CCR5 alleles could be observed in primary human CD4+ cells, providing selective advantage to CCR5^-/-^ double-mutant cells upon HIV1 infection *in vitro* and in an *in vivo* mouse model. Genotoxicity and specificity were assessed by the absence of DNA-repair protein foci formation, and by testing the frequency of induced mutagenesis at other potential cleavage sites. This work led to the opening in 2009 of a new clinical trial for AIDS patients, the first one involving a rare-cutting endonuclease. Recently, ZFN could also be used to inactivate CCR5 in human CD34+ cells [229]. These results provide a first step toward a therapeutic approach with the potential to create HIV resistant cells in all the blood cell lineages, instead of only within the T cell population.

### Virus Clipping

Nucleases targeted against viral sequences represent a novel class of antiviral agents that could cleave and either partially excise or completely eliminate viral DNA from infected cells, rendering them essentially “virus free”. This strategy mimics the cleavage of foreign DNA by restriction endonucleases in bacteria. The advantage of disrupting the viral genome, and not the various steps of viral replication, lies in the possibility of targeting latent forms of the virus. Latent viruses persist in the cell and are normally not affected by conventional treatments, which are effective only when the virus is actively replicating. This approach can be envisioned for different types of viruses, given that a double-strand DNA (dsDNA) stage is part of its lifecycle. Retroviruses, for example, constitute a large group of RNA viruses that upon entry into the target cell will generate by reverse transcription a dsDNA molecule that integrates into the host-cell genome. Other viruses that can be targeted by engineered meganucleases include the Polyomavirus, Papillomaviruses, Herpesvirus and Hepadnavirus, all of which belong to the group of DNA viruses and for which the genomic DNA remains as an episomal molecule in infected cells. Potential mechanisms are outlined in Fig. (**4**).

The feasibility of virus clipping is in the process of being validated by recent examples in the literature. For instance, it has been shown that ZFNs targeting Hepatitis B virus (HBV) DNA can efficiently cleave an HBV sequence present within a plasmid transfected in cultured cells [230]. Analysis of the DNA target reveals misrepair of affected HBV sequences within cultured cells coupled with a drop in pre-genomic viral RNA levels, an indicator of replication competence in cellular models that mimic HBV infection. Another promising approach lies in the prevention of viral infection using targeted meganucleases. Using a recombinant Herpes simplex virus (HSV-1) harboring an I-SceI restriction site, it was demonstrated that viral replication is impeded in COS-7 cells that have been transfected with a plasmid coding for the I-SceI meganuclease prior to infection (Smith *et al.* 2010, submitted). Furthermore, redesigned meganucleases derived from I-CreI were generated that are able to recognize and cleave sequences from the HSV genome. These HSV-specific meganucleases, when transfected prior to infection with a wild-type strain of HSV-1, reduce the levels of viral genomic DNA more than 50% compared to cells transfected with an empty vector. Still, while the above examples of virus clipping appear successful, efficacy remains to be assessed for integrated viral forms. Early experiments suggest that cleavage in both LTRs could result in viral genome loss, by tandem repeat recombination or rejoining of the two DSBs [130, 188, 197]. How these issues will be addressed remains to be seen.

## THE EFFICACY AND SAFETY ISSUES

In 2005, Urnov and colleagues created an engineered ZFN targeting the human IL2RG gene. By early 2010, a handful of diverse engineered nucleases able to edit human genes with high efficiency were described in the literature [16, 103, 105-108, 111, 178, 228]. One of these, a ZFN that can induce gene knock-out by NHEJ, is even in clinical trial [16]. These data reflect both the potential and limits of the technology as applied today. 

### Frequencies of Targeted Genome Modifications

In reviewing the different alternatives to viral gene transfer for gene therapy, it becomes clear that multiple factors can influence the success of each given approach. All targeted methods have aimed at reaching a symbolic “1% ratio” of targeted integration. Nevertheless, subtle variations such as reagents used, cell-types targeted, vector construction and even data analysis methods can have a significant impact on reported frequencies. Nucleases-based methods as discussed herein can achieve frequencies of >5% in immortalized cells, clearly indicative of the potential these approaches hold. However, a few recent studies suggest that these figures can strongly decrease in stem cells, which might represent one of the major hurdles for such technologies.

Lombardo and co-workers used a ZFN cleaving the human CCR5 gene to induce targeted insertion in different cell types [103]. Using non-integrative lentiviral vectors instead of transfection, up to 50% of targeted events were observed in K562 and Jurkat cells, about 5% in ES cells, and close to 0.1% in CD34+ cord blood progenitor cells [103]. However, Zou and colleagues observed lower figures in human ES cells [108]. Using ZFNs targeting a GFP reporter gene, they obtained frequencies in the range of 3% in 293T cells, but only of 0.1-0.2% in hES and iPS. With a tailored ZFN targeting the PIG-A human gene, the same authors observed even lower frequencies, in the range of 2-4x10^-4^ for human ES cells, and 10^-5^ for human iPS. In another study, Hockemeyer *et al.* used a selection process to identify targeted recombination events induced by tailored ZFNs in the human OCT4, AAVS1 and PITX3 endogenous genes in human ES and iPS cells. High frequencies of targeted integrations were observed among transformants (up to 61%), but the absolute rate of gene targeting is not indicated [107].

Discrepancies between the studies of Lombardo and colleagues and those of Zou and coworkers in the observed targeted recombination frequencies in hESCs could be due to the methods used, the quality of the nuclease employed, or even the cell type. In any case, efficacies in stem cells seem to be far below what can be achieved with viral vectors, or even non-viral methods such as those utilizing enhanced versions of the Sleeping Beauty transposon [70].

One hypothesis to explain the low frequencies of HGT events observed by Lombardo *et al.* in CD34+ cells, and by Zou *et al.* in human ES and iPS cells, is that these cells would be less proficient for HR. In principle, endonuclease-mediated mutagenesis should be less limiting than HR-based approaches, for NHEJ is not limited to the S/G2 phases of the cell cycle. Nevertheless, lower efficacies can result from factors other than a limiting repair pathway. The induction of indels by endonucleases has been reported for several cell types and organisms, often with impressive efficacies: the CCR5 gene could be mutated with frequencies in the range of 30-54% in primary T-cells using an adenoviral vector expressing a ZFN [16]. Zou *et al.* also monitored the frequency of targeted mutagenesis induced by their PIG-A ZFN, and observed only 4.6x10^-6^ of indels in ES cells. This frequency, about 100 times lower than the frequency of DSB-induced HGT in the same cells, has to be considered cautiously, for it was monitored by a selection process. However, it suggests that the limiting factor in hES cells could actually be suboptimal vectorization and/or lower expression levels of the endonuclease, which would affect both HGT and targeted mutagenesis.

These data emphasize the need for a more complete body of data regarding targeted recombination in stem cells, and likely reflect the inherent difficulties in handling these cells, regardless of the mechanism of action (targeted mutagenesis or HGT) of the nuclease.

### Specificity

Whereas the efficacy of targeted approaches can be measured in terms of directed events (e.g. percent mutagenesis at the targeted locus), a true benchmark for specificity remains elusive as the very nature of monitored events can vary by assay. For instance, the detection of repair protein foci (phosphorylated H2AX or 53BP1) is routinely used to measure nuclease toxicity [16, 105, 178, 191, 231, 232]. The high levels of repair foci that occur naturally in immortalized cells, however, make this assay relatively insensitive despite the assayed proteins being highly specific. A more straightforward assay using immortalized cells involves monitoring cell survival in the presence of increasing doses of nuclease to compare the relative toxicity of different endonucleases [106, 178, 232, 233]. This assay in turn suffers from being hardly predictive of what will happen in true target cells, wherein expression will likely be more limited. Endonuclease-induced indels have also been a major concern and several studies have described the characterization of mutagenesis in promiscuous sequences, typically found to be null or infrequent [16, 107]. However, targeting the CCR5 gene with a dedicated ZFN induced significant mutagenesis in the related CCR2 gene (4-5% for CCR2 versus 36% for CCR5) [16] . Furthermore, studies showing that the occurrence of several DSBs can result in translocation have raised additional concerns [234, 235]. The toolbox for characterizing potential genotoxicity certainly needs to be upgraded to address these issues, and several efforts are currently underway.

A number of standard quality control tests can be used to test the status of stem cells, *e.g.* monitoring (i) growth rate; (ii) karyotype; (iii) cell morphology; (iv) the expression of genes marking the differentiation status, and; (v) multipotency (differentiation tests *in vitro* or *in vivo*). Such tests have been used *after* stem cell engineering with nucleases [107, 108], but also with other reagents [70], without detection of any gross alteration. Finally, using a humanized mouse model to study the fate of engineered cells [16] appears effective for assessing both activity and safety.

## CONCLUSION

The difficulties in harnessing the potential of endonuclease-based targeted approaches can best be placed in perspective when one considers that the first clinical trial using these methods involves a gene knock-out technique. The purpose of replacing current methods that use random transgene insertion by those using targeted recombination is to have better control over the resulting engineered cells. More specifically, the goal is not only to promote and dictate transgene expression, but also to avoid adverse events such as insertional mutagenesis. In addition, these benefits need to be significant. For example, whereas retroviral gene transfer in 20 patients treated for X-SCID [3, 5] and 2 patients treated for CGD [6] resulted in several cases of leukemia (X-SCID) or myoplasia (CGD), hundreds of patients have received T-cells engineered with similar vectors [236] without a single occurrence of induced malignant transformation. In these cases the integration sites were different from those commonly found in HSCs, perhaps explaining the absence of SAEs [237]. Thus, safety concerns can vary depending on the indication.

The benefits of targeted methods must also not be at the expense of new or unexpected significant drawbacks. In the case of endonuclease-mediated recombination, the obvious potential drawbacks are (i) a potential genotoxicity that remains to be assessed, and; (ii) lower efficacy. Genotoxicity is an inherent problem of enzymes that act on nucleic acids, though one can expect that highly specific endonucleases would reduce or abolish this issue. The lower efficacy, on the other hand, stems from limitations in the levels of homologous recombination within the cells, an obstacle underscored by experiments in stem cells and especially in HSCs. Several studies aspire to alleviate this tissue-specific variability by using iPS cells, although it not clear if (a) they display better recombination rates or; (b) they will be acceptable for cell therapy in the short term.

Currently, random insertion using a quality vector (preferentially a viral one) is more efficient than the highest reported targeted-insertion rate, regardless of the cell type. For any emerging method it is therefore important to strive for both an excellent recombination rate as well as a low threshold in terms of required activity. Whereas the activity level depends on the cell type (for HR proficiency) and vectorization (to bring enough nuclease and repair matrix into the target cell), the threshold of required activity rather depends on the indication. The real question is whether the efficacies described above represent a limit. Differences in efficacy could be less stringent for indications such as haemophilia, wherein expression of 1% of wild-type levels is sufficient to result in a therapeutic effect [238]. If gene correction is associated with a selective advantage (e.g. SCID diseases), a low frequency of repair should be enough to provide a therapeutic effect [89]. Then again, SCID has to be addressed in early progenitor cells, which could prove especially difficult to target. Whether these limitations also apply to the same extent for gene knock-out based strategies is unclear. Although the CCR5 clinical trial cannot provide a benchmark for strategies based on targeted insertion, the absence of toxicity reported so far is an important clue for nuclease-based approaches. Pioneering studies using a good safe harbor/nuclease coupled with one of the more “accessible” diseases (such as the ones quoted above) could represent an essential milestone, answering many important questions. In this respect, the choice of a good nuclease (high activity/toxicity ratio) targeting a good safe-harbor locus (stable expression and no impact on neighboring genes) will be extremely important. However, the choice of the target cell might be at least as important as the mechanism of action of the nuclease: recent publications have shown that the use of stem cells is associated with potential additional difficulties, and it should be noted that the current CCR5 clinical trial is based on the treatment of T cells, and not of HSCs (the possibility to treat HSCs is being currently investigated [229]). Furthermore, one can expect that a humanized mouse model will be key in deciding whether currently achievable recombination rates in stem cells will be sufficient.

For endonuclease-based approaches, proper expression of the nuclease within the target cell is certainly one of the most important aspects for both efficacy and specificity. A good efficacy relies on the introduction of a sufficient amount of nuclease into the target cell. Although the off-site cleavage of these molecules has been significantly reduced by a variety of strategies, a low level of activity over an extended period of time could have significant consequences. Several methods of vectorization have been used for the generation of nuclease mediated modification events in human cells, including non viral methods (electroporation) and the use of viral vectors (AAV vectors, integration-defective lentiviral vectors (IDLV) and adenovirus vectors, the latter solution being used in the CCR5 clinical trial). Nevertheless, one of the biggest advantages of nuclease-based approaches could be that they could alleviate the need for viral vectors, which represent a real hurdle in terms of GMP manufacturing. In this regard, nuclease-based strategies are  more in competition with transposon-based (targeted or non-targeted) rather than with viral vector-based approaches.

## Figures and Tables

**Fig. (1):**
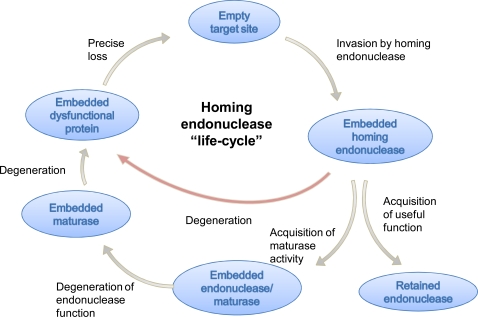
Proposed life-cycle of a homing endonuclease (see text for details). Adapted from [145].

**Fig. (2):**
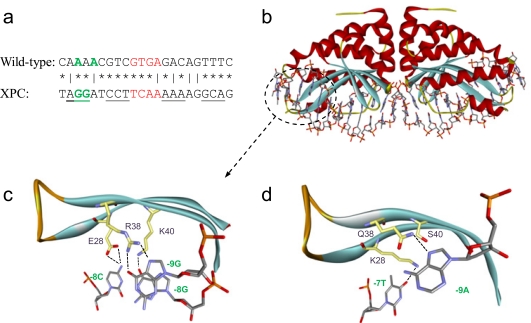
Re-engineering meganuclease specificity: example of a custom meganuclease targeting a sequence from the human XPC gene. (**a**) Comparison of the starting and final targets. (**b**) Crystal structure of the engineered I-CreI variant in complex with target DNA. Details of protein-DNA contacts from the encircled region are highlighted (**c**) to demonstrate how a clustered approach leads to significant changes in specificity contacts. The native protein-DNA contacts for I-CreI (**d**) are shown for reference. Adapted from [161].

**Fig. (3):**
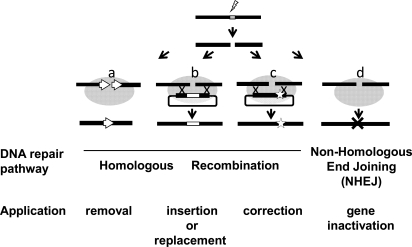
Endonuclease-induced gene targeting approaches. Upon cleavage, DNA repair mechanisms may result in one of several outcomes. When a double-strand break is targeted between two direct repeats (**a**), homologous recombination can result in the deletion of one repeat together with the intervening sequence. Gene insertion (**b**) or correction (**c**) can be achieved by the introduction of a DNA repair matrix containing sequences homologous to the endogenous sequence surrounding the DNA break. Mutations can be corrected either at or distal to the break, with the frequency of correction decreasing with increasing distance. The misrepair of DNA ends by error-prone non-homologous end joining (**d**) can result in insertions or deletions of various sizes, leading to gene inactivation.

**Fig. (4):**
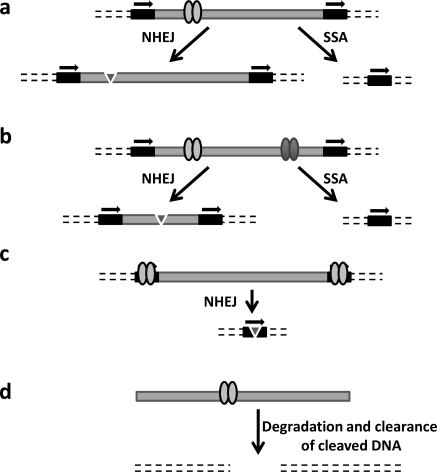
Meganucleases as antiviral agents. Pathways by which viral sequences belonging either to essential genes or regulatory regions can be inactivated are shown. Gene inactivation can result from small insertions/deletions that introduce lethal mutations in the viral genome by error-prone non-homologous recombination (panel **a**). Large deletions can be introduced by DNA cleavage and repair when using two different meganucleases targeting the same viral genome at different positions (panel **b**), or by rejoining DNA ends when cleavage occurs in a repeated region of the viral genome (panel **c**). Alternatively, when cleavage occurs between two direct repeats (e.g.: the LTR retroviral sequences), deletion of the intervening sequences can be generated by tandem repeat recombination (SSA, panels **a** and **b**). While the pathways depicted in panels a-c are valid for integrated as well as episomal viruses, the latter can also be targeted via the degradation and clearance of the viral genome upon DNA cleavage (panel **d**).

## ABBREVIATIONS

AAV: = Adeno-Associated Virus
DSB: = Double-Strand Break
ES: = Embryonic Stem (cells)
HGT: = Homologous Gene Targeting
HR: = Homologous Recombination
HSC: = Haematopoietic Stem Cell
iPS: = Induced Pluripotent Stem (cells)
NHEJ: = Non-homologous End Joining
SCID: = Severe Combined Immune Deficiency
SFHR: = Short Fragment Homologous Replacement
TALE: = Transcription Activator-Like Effector
TFO: = Triplex-Forming Oligonucleotides
ZFP: = Zinc-Finger Protein
ZFN: = Zinc-Finger Nuclease
